# Surgical versus expectant management in women with an incomplete evacuation of the uterus after treatment with misoprostol for miscarriage: the MisoREST trial

**DOI:** 10.1186/1471-2393-13-102

**Published:** 2013-05-02

**Authors:** Marianne AC Verschoor, Marike Lemmers, Patrick M Bossuyt, Giuseppe CM Graziosi, Petra J Hajenius, Dave J Hendriks, Marcel AH van Hooff, Hannah S van Meurs, Brent C Opmeer, Maurits W van Tulder, Liesanne Bouwma, Ruby Catshoek, Peggy Geomini, Ellen R Klinkert, Josje Langenveld, Theodoor E Nieboer, J Marinus van der Ploeg, Celine M Radder, Taeke Spinder, Lucy F van der Voet, Ben Willem J Mol, Judith AF Huirne, Willem M Ankum

**Affiliations:** 1 Department of Obstetrics and Gynaecology, Academic Medical Centre, Amsterdam, The Netherlands; 2 Department of Obstetrics and Gynaecology, Amphia Hospital, Breda, The Netherlands; 3 Department of Obstetrics and Gynaecology, Flevo Hospital, Almere, The Netherlands; 4 Department of Obstetrics and Gynaecology, VU Medical Centre, Amsterdam, The Netherlands; 5 Department of Clinical Epidemiology, University of Amsterdam, Amsterdam, the Netherlands; 6 Department of Obstetrics and Gynaecology, St. Antonius Hospital, Nieuwegein, the Netherlands; 7 Department of Obstetrics and Gynaecology, St. Franciscus Hospital, Rotterdam, The Netherlands; 8 Clinical Research Unit, Academic Medical Centre, Amsterdam, the Netherlands; 9 Department of Health Sciences & EGMO + Institute for Health and Care Research, Faculty of Earth and Life Sciences, VU University, Amsterdam, The Netherlands; 10 Department of Obstetrics and Gynaecology, Jeroen Bosch Hospital, ‘s Hertogenbosch, the Netherlands; 11 Department of Obstetrics and Gynaecology, Maastricht University Medical Centre, Maastricht, the Netherlands; 12 Department of Obstetrics and Gynaecology, Maxima Medical Centre, Veldhoven, the Netherlands; 13 Department of Obstetrics and Gynaecology, University Medical Centre Groningen, Groningen, the Netherlands; 14 Department of Obstetrics and Gynaecology, Atrium Medical Centre, Heerlen, the Netherlands; 15 Department of Obstetrics and Gynaecology, Radbout University, Nijmegen Medical Centre, Nijmegen, the Netherlands; 16 Department of Obstetrics and Gynaecology, Martini Hospital, Groningen, the Netherlands; 17 Department of Obstetrics and Gynaecology, St Lucas Andreas Hospital, Amsterdam, the Netherlands; 18 Department of Obstetrics and Gynaecology, Medical Centre Leeuwarden, Leeuwarden, the Netherlands; 19 Department of Obstetrics and Gynaecology, Deventer Hospital, Deventer, the Netherlands

**Keywords:** Curettage, Expectant management, Incomplete evacuation, Miscarriage, Misoprostol

## Abstract

**Background:**

Medical treatment with misoprostol is a non-invasive and inexpensive treatment option in first trimester miscarriage. However, about 30% of women treated with misoprostol have incomplete evacuation of the uterus. Despite being relatively asymptomatic in most cases, this finding often leads to additional surgical treatment (curettage). A comparison of effectiveness and cost-effectiveness of surgical management versus expectant management is lacking in women with incomplete miscarriage after misoprostol.

**Methods/Design:**

The proposed study is a multicentre randomized controlled trial that assesses the costs and effects of curettage versus expectant management in women with incomplete evacuation of the uterus after misoprostol treatment for first trimester miscarriage.

Eligible women will be randomized, after informed consent, within 24 hours after identification of incomplete evacuation of the uterus by ultrasound scanning. Women are randomly allocated to surgical or expectant management. Curettage is performed within three days after randomization.

Primary outcome is the sonographic finding of an empty uterus (maximal diameter of any contents of the uterine cavity < 10 millimeters) six weeks after study entry. Secondary outcomes are patients’ quality of life, surgical outcome parameters, the type and number of re-interventions during the first three months and pregnancy rates and outcome 12 months after study entry.

**Discussion:**

This trial will provide evidence for the (cost) effectiveness of surgical versus expectant management in women with incomplete evacuation of the uterus after misoprostol treatment for first trimester miscarriage.

**Trial registration:**

Dutch Trial Register: NTR3110

## Background

Miscarriage is a frequent complication in first trimester pregnancy which occurs in 10-15% of pregnant women and results in between 18.000 and 27.000 miscarriages in The Netherlands each year [1]. In the past, Dutch women diagnosed with miscarriage were either managed expectantly, with complete expulsion of the products of conception knowing to occur within two weeks in 50% of women, or were offered surgical management (curettage) as an alternative option [2,3]. Only recently, medical treatment with misoprostol has been introduced as an outpatient and inexpensive treatment option, which is available on demand and easy to use. Treatment with misoprostol is effective in 50-80% of women with miscarriages [4-11]. Initial treatment with misoprostol seems to be more cost-effective than immediate curettage, especially in specific subgroups of women [12-15]. There are no significant differences between the use of misoprostol versus curettage on pelvic infections or ongoing pregnancies during follow-up [16].

According to a national survey in 2010, about 50% of Dutch hospitals use misoprostol in the management of miscarriage, and this figure probably underestimates its current use [17]. Misoprostol is currently applied in about 10,000 women with miscarriages each year in The Netherlands.

A problem in the treatment with misoprostol is that approximately one third of women show incomplete evacuation on ultrasound scanning during follow-up, despite being relatively asymptomatic [18,19]. Although misoprostol is used ever more frequently, the sonographic image of incomplete evacuation still leads to additional surgery, i.e. curettage.

We estimate that surgical management is applied in approximately 3,000 of these women who, despite having experienced a clinically successful miscarriage after misoprostol administration, are left with incomplete evacuation as diagnosed sonographically. If expectant management would turn out to be (cost)effective as compared to curettage, this could potentially save 3.000 surgical procedures -i.e. curettages.

There is no doubt that curettage is an effective treatment for women with an incomplete miscarriage, but it also includes the risk on perforation and the forming of intra-uterine adhesions, in particular in women with a previous curettage. It is unknown whether curettage is also cost effective in relatively asymptomatic women with sonographic evidence of incomplete evacuation after initial misoprostol treatment for miscarriage.

A comparison of surgical management versus expectant management is still lacking and data on the costs of both strategies are not available.

## Methods/Design

### Objective

The objective of this study is to compare the (cost) effectiveness of surgical versus expectant management in women with sonographic evidence for incomplete evacuation of the uterus after misoprostol treatment for first trimester miscarriage.

### Participants/eligibility criteria

All women > 18 years with sonographic evidence of incomplete evacuation of the uterus, one to two weeks after having been treated initially with misoprostol for a first trimester miscarriage, are informed on the trial. They can participate in the trial when they have an intra-uterine remnant or an anterio-posterior diameter of the uterine cavity of ≥ 10 millimeters. Exclusion criteria are severe vaginal bleeding or severe abdominal pain that needs acute intervention, fever (> 38.0 Celcius) or sepsis requiring antibiotic treatment and curettage, contraindications for curettage, or a failed misoprostol-induced miscarriage with an intact gestational sac still in utero.

### Procedures, recruitment, randomization, collection of baseline data

The study is a multicentre randomized controlled study. The study is performed within the Dutch Consortium for Studies in Women’s Health and Reproductivity (http://www.studies-obsgyn.nl). Participating hospitals can be district, teaching or third referral hospitals. 25 Hospitals are participating at this moment, and we expect participation of at least five more hospitals.

Before study entry, women are informed about the aims, methods, reasonably anticipated benefits and potential hazards of the study. They are informed that their participation is voluntary and that they may withdraw consent to participate at any time during the study. Choosing not to participate will not affect care. An independent gynecologist is available. Women are asked to decide whether or not to participate in this study within 24 hours after counselling. Written informed consent is obtained. The consent form must be signed before performance of any study-related activity. Eligible patients are randomly allocated to either curettage within 3 workdays (experimental strategy) or expectant management (control strategy). Randomisation is performed through a web-based database located in the central data collection unit in the Academic Medical Centre in Amsterdam. Patients who refuse to participate in the trial are asked to take part in the observational arm of the study. Due to the nature of the two treatment options blinding will not be possible.

Baseline demographic, obstetric and medical histories are recorded for all women, including number of previous miscarriages and curettages. Gestational age and transvaginal findings at misoprostol use in the index pregnancy and endometrial thickness at randomization are registered.

All details of treatment and follow-up are recorded in a case record form that is accessible for authorised research personnel through a website http://www.studies-obsgyn.nl/misorest.

### Interventions and follow up

**Figure 1:**
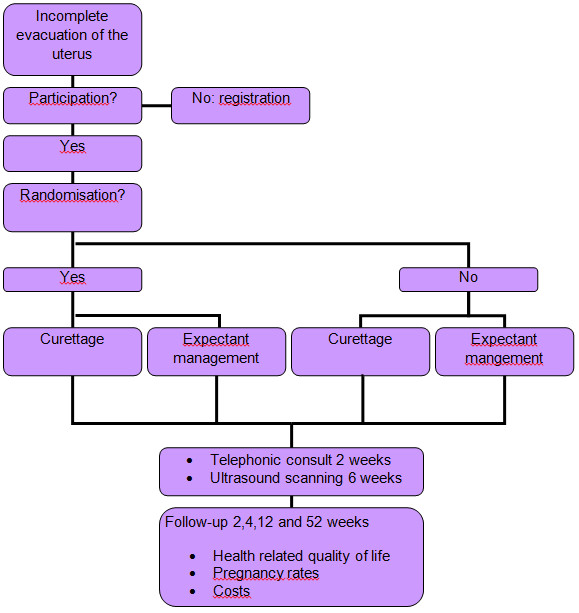
**Flowchart. **MisoREST trial: randomisation and follow-up.

Women allocated to surgical management are scheduled for curettage under general, regional or local anesthesia in a day-care setting within three days after randomization. Transvaginal sonographic examination is scheduled six weeks after curettage (Figure 1).

Women allocated to expectant management receive no further treatment. There is a consultation by telephone two weeks after randomization by a research nurse. Transvaginal sonographic examination is scheduled six weeks after randomization. In case of severe complaints, defined as heavy vaginal bleeding or severe abdominal pain, or the occurrence of fever (> 38.0 Celcius) an emergency curettage will be performed.

In both groups, in case of persisting abnormalities at sonographic examination six weeks after randomization, women will be scheduled for (re-)curettage.

### Outcome measures

The main outcome measure is the sonographic finding of an empty uterus (maximal diameter of any contents of the uterine cavity < 10 millimeters) six weeks after study entry/randomization.

Secondary outcomes are patients’ quality of life, measured with validated questionnaires at baseline (before randomization), 1–2 weeks, 4 weeks and 3 months after randomization; surgical outcome parameters; the type and number of any re-interventions during the first three months; pregnancy rates 12 months after study entry, with the recording of unexposed cycles and cycles in which contraceptives were used; and costs.

Questionnaires used for Quality of Life measurement are the Hospital Anxiety and Depression Scale (HADS), the Short Form Health Survey (SF-36), Recovery Index (RI-10) and EuroQol Health Questionnaire (EQ-5D). At baseline HADS, RI-10, EQ-5D will be registered four weeks after study entry, HADS, SF-36 and RI-10 will be completed. 12 months after study entry patients will be asked to fill in a questionnaire about their desire for future pregnancy and the occurrence and outcome of a next pregnancy. In case the patient does not return the questionnaires, a reminder is sent.

### Economic evaluation

The economic evaluation will be performed alongside the clinical trial and from a societal perspective. It is designed as a cost-effectiveness analysis of surgical management as compared to expectant management, estimating the costs per patient with a complete evacuation of the uterus as primary outcome. All relevant costs will be measured, such as direct medical costs (health care consumption), direct non-medical costs (informal care) and indirect costs (costs of production loss).

Volumes of health care resource use are measured prospectively using the case record form. Health resource use outside the hospital is recorded by questionnaires filled out by the patients 3 months after randomization. The Health and Labor Questionnaire is used to document absence from paid work [20].

This evaluation will provide insight on whether expectant management in women with incomplete evacuation of the uterus after misoprostol treatment for miscarriage will reduce costs as compared to surgical management.

### Statistical issues

#### Sample size

The primary study question is phrased as a superiority hypothesis of surgical management versus expectant management. Surgical management is expected to lead to a higher proportion of patients successfully treated, but we anticipate that women will prefer expectant management if the proportion of success for curettage is not substantially higher. Anticipating a 98% success rate with curettage, versus 85% for expectant management, we would need to randomise 130 patients on a 1:1 basis. Assuming a drop-out rate of 20%, we plan to include 162 patients in total (81 per arm).

A one-sided 95.0% confidence interval for a ln (relative risk) expected to be 0.142 will extend 0.091 from the observed ln(relative risk) (corresponding to confidence limits of 1.053 and/or 1.262 for a relative risk of 1.153).

#### Data analysis

Data will be analyzed according to the intention to treat principle. Within each group, we will calculate the proportion of randomized women with an empty uterus, as verified by ultrasound scanning. The effectiveness of surgical management versus expectant management is expressed as a relative risk, with 95% confidence interval.

Univariate and multivariate logistic regression analyses are used to analyse the relative contribution of findings on ultrasound versus other predictive factors for complete evacuation on misoprostol treatment, like age, parity and gestational age at initial treatment.

A linear mixed model is used for comparison of the study groups on SF-36 and HADS summary scores while accounting for the baseline values.

Differences in pregnancy rates 12 months after study entry in both study groups will be compared using Kaplan Meier curves and tested with the log-rank test, thereby censoring for unexposed cycles and for cycles where contraceptives were used.

The economic analysis will be done according to the intention-to-treat principle. Missing data will be imputed using multiple imputation techniques. We will compare the differences in total costs between the two interventions to difference in effect, expressed as the costs per completely cured patient (complete evacuation of the uterus six weeks after randomization). We will also perform a cost-utility analysis based on the EQ-5D. Bootstrapping will be used for pair-wise comparison of the mean differences in total costs. Cost-effectiveness and cost-utility ratios will be estimated using bootstrapping techniques and graphically presented on cost-effectiveness planes and cost-effectiveness acceptability curves.

#### Data safety monitoring committee

Serious Adverse Events (SAE’s) will be reported to a Data Safety Monitoring Committee (DSMC). The DSMC can order to perform an interim analysis and, if indicated, terminate the trial prematurely.

#### Interim analysis

Because of the relatively small sample size and the expected duration of inclusion no interim analysis will be performed. This was approved by the DSMC. The study group has already completed studies comparing curettage versus expectant management and studies comparing misoprostol versus curettage in women with a first trimester miscarriage. No serious adverse events have been recorded within these studies.

#### Ethical consideration

This study is approved by the National Central Committee on Research involving Human Subjects (CCMO - NL38637.018.11), by the ethics committee of the Academic Medical Centre Amsterdam (Ref. No. 11–373) and by the boards of management of all participating hospitals.The trial is registered in the Dutch Trial Register (NTR3110, http://www.trialregister.nl).

## Discussion

Recently, medical treatment with misoprostol was introduced as a non-invasive and cost effective treatment option in women with first trimester miscarriage. However, approximately 30% of women show incomplete evacuation of the uterus on ultrasound scanning during follow-up. This still leads to additional surgery in a substantial part of women receiving misoprostol, i.e. curettage, although most women are relatively asymptomatic.

This study is designed to compare the (cost) effectiveness of surgical management versus expectant management in women with sonographic evidence of incomplete evacuation of a miscarriage after primary misoprostol treatment. To our knowledge there are no other ongoing trials in the Netherlands or other countries evaluating this subject. It is anticipated that the outcome of this study can save up to 3000 surgical procedures in The Netherlands yearly.

## Abbreviations

HADS: Hospital Anxiety and Depression Scale; SF-36: Short form health survey; RI-10: Recovery index; EQ-5D: EuroQol Health Questionnaire; SAE’s: Serious adverse events; DSMC: Data safety monitoring committee.

## Competing interests

The authors declare that they have no competing interests.

## Authors’ contributions

WA, PB, PG, PH, MvH, JH, BWM, BO and MvT were involved in the conception and design of the study. MV, ML, PH, MvH, JH, MvT, BWM and WA drafted the manuscript. All authors mentioned in the manuscript are members of the MisoREST-trial study group. They are local investigators at the participating centres. All authors read, edited and approved the final manuscript.

## Pre-publication history

The pre-publication history for this paper can be accessed here:

http://www.biomedcentral.com/1471-2393/13/102/prepub
